# Asilomar Goes Underground: The Long Legacy of Recombinant DNA Hazard Debates for the Greater Boston Area Biotechnology Industry

**DOI:** 10.1007/s10739-025-09806-x

**Published:** 2025-03-07

**Authors:** Robin Wolfe Scheffler

**Affiliations:** Program in Science, Technology, and Society, MIT, 77 Massachusetts Avenue E51-186, Cambridge, MA 02139 USA

**Keywords:** Recombinant DNA, Biomanufacturing, Asilomar, Regulation, Biotechnology, Credit

## Abstract

In 1975, a meeting on the potential hazards of recently invented recombinant DNA techniques was held at the Asilomar Conference Center in California. This meeting gave rise to a global debate over the safety and regulation of recombinant DNA (rDNA). In this paper, I use the historical development of recombinant DNA regulation in the Greater Boston Area—now home to the densest cluster of the biotechnology industry in the world—to provide a different interpretation of the legacies of Asilomar. While most accounts of Asilomar have considered its brief and dramatic impact on molecular biology on a national scale, an equally meaningful and overlooked impact is to be found in the development of regulations around recombinant DNA at the local level. Rather than hindering research, these events enabled the operations of the modern commercial biotechnology industry, which was founded on the promise of recombinant DNA. This approach highlights a different legacy of Asilomar, one which did not end with expert consensus that recombinant DNA was safe. Instead, attending to the material, infrastructural aspects of working with recombinant DNA in commercial settings reveals a wide range of communities involved in determining the social impacts of Asilomar—communities asking a broader set of questions about recombinant DNA than those originally posed in 1975.

## Introduction: Following Asilomar Down the Drain

On June 4, 1982, a technician at Biogen’s Cambridge, Massachusetts laboratory poured five gallons of *E. coli* bacteria genetically engineered using recombinant DNA (rDNA) down a sink. The release of the bacteria caused a crisis for the biotechnology firm. Following the 1975 International Conference on Recombinant DNA Molecules at Asilomar, the City of Cambridge had gained global notoriety for contemplating banning the use of rDNA outright, and then for the rigor of its municipal rDNA regulations (Krimsky 1982a, pp. 298–306; Mendelsohn 1984; Durant 2010; Botelho 2021). In the spring of 1982, Biogen was engaged in negotiations with the city to develop a pilot biomanufacturing plant that would cultivate rDNA-modified bacteria at a scale 100 times greater than the amounts allowed under the city’s regulations. The technician’s error release revived dormant concerns for rDNA’s safety and threatened to derail the firm’s expansion. Biogen reassured the public that the bacteria were “not a dangerous organism.” Nonetheless, the president of Biogen found himself sampling water from sewer drains near the laboratory and answering pointed questions from city officials (Luberoff 1982).

This moment, which I encountered during my ongoing research into the history of the biotechnology industry in the Greater Boston Area (GBA), offers a point of departure for reconsidering how we should approach the legacy of Asilomar on its fiftieth anniversary. Asilomar, a meeting of scientists, journalists, and legal experts convened to consider the potential hazards of recombinant DNA research and the best strategies for their mitigation, has become a touchstone for subsequent science policy discussions (Parthasarathy 2015; Campos 2024). Historians and sociologists of science have focused on the national debate over the regulation of rDNA research that followed this conference, which was dominated by academic molecular biologists and their patrons in the federal government. Viewed through the prism of these debates, the most important consequences of Asilomar were those events that did not come to pass: despite widespread calls for further civilian control of rDNA, no federal regulations were imposed on the technique and oversight of rDNA remained within the scientific community (Wright 1994; Fredrickson 2001). Even in the view of knowledgeable commentators involved in the Cambridge debates, discussions of rDNA regulation at the local level were quickly superseded and deflated by this national trend (Krimsky 1982a, p. 311). Subsequent accounts have continued to focus on national regulatory regimes for rDNA and other biotechnologies (Gottweis 1998; Jasanoff 2005).

Following national events, however, provides a poor means of apprehending Asilomar’s ongoing impact on the relationship between biotechnology and society. While questions of rDNA governance might have seemed resolved at the federal level by the early 1980s, this decade and those which followed were marked by dynamism at the local level. Within the GBA dozens of communities followed Cambridge in passing their own regulations on rDNA—ninety by 2024—and during these decades the GBA became host to the densest and liveliest cluster of biotechnology research in the world.^1^ These regulations, however, arose in response not to academic rDNA experiments but to commercial rDNA applications, which soon accounted for 95% or more of the technique’s use.^2^ Although commercial uses of rDNA rapidly came to dominate regulatory attention, historians of biology have continued to default to perspectives on its regulation voiced by university-based molecular biologists. However, the settled nature of discussions over the regulation of academic rDNA research obscures the fact that the emergence and expansion of commercial rDNA frequently revived the issues of Asilomar as biotechnology moved to new communities.

Following private biotechnology companies as they sought sites for their rDNA work indicates that a much broader range of actors and institutions participated in shaping the legacy of Asilomar. Although most commentators have been quick to characterize ongoing concerns about genetic engineering as an unfortunate public overreaction to Asilomar, these concerns were nevertheless a constant and consequential presence in how firms and their backers assessed potential locations. Experimental work in the life sciences requires multiple forms of external support that tie the operation of laboratories to their physical and social surroundings.^3^ In contrast to established universities, fledgling biotechnology companies faced the task of creating and maintaining infrastructure for rDNA research and manufacturing from the ground up—from obtaining financing and construction permits to gathering materials and disposing of waste. These negotiations left them far more exposed to the concerns of their host communities than academic institutions. While private companies did not receive federal grant support and were thus not constrained by federal guidelines on rDNA use, they were acutely sensitive to the dynamics of what Tiffany Nichols (2024) has called the “expanded laboratory environment,” and particularly to local regulation. These challenges, and the promises of wealth from new biotechnological innovations, drew politicians, investors, bankers, and citizens into an ongoing conversation about how the questions raised at Asilomar should be answered.

The legacy of Asilomar as it was received, refracted, and debated by communities and businesses around the GBA looks very different from the picture at a national level, as do the lessons we may draw from it regarding the relationship between society and emerging technologies. Science studies scholars have followed the public-facing rhetoric of businesses and academic scientists during the 1980s in assuming that external regulation was a threat to the progress of scientific research and that self-regulation was preferable. Even critics of how Asilomar was resolved, such as Susan Wright (1994), nonetheless assumed that scientists would benefit from light regulation. The relaxation of federal regulations was part and parcel of the deregulatory, pro-business impulse of the Reagan Administration (Weiner 1999, pp. 297–298). However, conflating academic and business regulation may have been misleading. Starting in the early 1990s, economists seeking to understand the nature of industry clusters suggested that government regulations, even those that appeared adverse at first glance, could promote growth rather than hinder it (Porter and van der Linde 1995). More recently Maryann Feldman has suggested that local regulations were an important, rather than incidental, feature of biotechnology’s growth in the GBA. While geographers of innovation typically speak of the anchoring effects of businesses or academic institutions, local regulations may have had a similar anchoring impact on biotechnology—a claim anticipated by officials working in cities such as Cambridge (Lipson 2003; Lowe and Feldman 2008).

Local regulations mediated between the material and financial needs of biotechnology firms and unpredictable public concerns regarding genetic engineering propagated by Asilomar. Biotechnology firms and economic developers turned to local regulations as a means of generating a stable environment for investment in research and biomanufacturing infrastructure. Thus the legacy of Asilomar was not only defined through national consensus or expert judgement but also through the outcomes of myriad and prolonged local interactions among cities, citizens, biotechnology firms, nonprofits, and developers. The first part of this article traces the novelty and force of Cambridge-based activists’ reframing of rDNA as a matter of urban public health rather than just one of national research policy, which brought Asilomar into the realm of municipal politics and expanded the range of individuals involved in deliberations. The second part illustrates how and why the first biotechnology companies in the GBA came to see local regulation as beneficial rather than inhibitory as they contemplated using rDNA in biomanufacturing and research. This shift involved company leadership appreciating the virtues of research in Cambridge, where MIT and Harvard had already borne the brunt of the rDNA debate, as well as the vices brought by the absence of regulations in neighboring communities. The final section examines how public health regulations for rDNA were incorporated into the financial infrastructure of the industry, turning regulations intended to govern the risk of the technology itself into a means of managing the business risks of public intervention.

## Localizing Asilomar: rDNA as a Public Health Hazard

Although the Cambridge rDNA discussions were not the first case of a municipal effort to regulate rDNA (that distinction belonged to Ann Arbor, Michigan), they became the most energetic and visible (Wright 1994, p. 190). Cambridge acted just as scientists appeared to be reaching consensus. Concern for the safety of rDNA reached a peak within the molecular biology community in 1974, when many members endorsed a voluntary deferral of some potentially hazardous experiments; when Asilomar convened in 1975, its emphasis was not on whether rDNA research should occur but under what conditions it should proceed—although some scientists not at Asilomar dissented from this goal. These discussions aimed at establishing a national framework for research, but just as the NIH issued its guidelines for rDNA research in June of 1976 (based largely on the Asilomar discussions), Asilomar-related debates among Boston-area molecular biologists spilled over and prompted the Cambridge City Council to consider its own regulations for rDNA. These civic deliberations are most often memorialized as a signature moment of confrontation between scientists and the public, but their more subtle but enduring impact was to place a new frame over how the hazards of rDNA research could be governed—as a matter of urban public health rather than national research policy. This reframing placed the relationships between laboratories and their host communities at the forefront of the conversation, with enduring consequences for how Asilomar would shape the uses of rDNA.

For rDNA to become a matter of public health, Cambridge officials needed a new understanding of the relationship between laboratories and their surroundings. The approach that emerged from Asilomar focused not on binary divisions between safety and danger but on a multifactorial system for categorizing and managing risks and weighing these risks against forecasted benefits (Berg et al. 1974, 1975; Krimsky 1982a, pp. 381–385). Herbert Gottweis (1998) has argued that this system was adept at allowing for technical and rhetorical critiques of rDNA’s safety while allowing molecular biologists to remain the central actors in its regulation. The management of risk divided the world into spaces within and without laboratories where rDNA work would take place and kept their management firmly within the province of experimental operations; there was no suggestion that local communities would be involved in the management of rDNA work. Following Asilomar, the formulation of the first guidelines for rDNA research by the NIH’s Recombinant DNA Advisory Committee (NIH RAC), composed primarily of professional biomedical researchers, maintained this framing as it drafted principles for NIH-funded researchers in 1975–1976. Hazards were ranked and managed through processes of either physical containment—from P1, the least restrictive, to P4, the most restrictive—or through biological containment by using specific strains of bacteria or viruses deemed unlikely to spread to humans.^4^

The framework of risk management offered at Asilomar was the target of spirited critique from the outset, and threads of this critique provided avenues for reframing rDNA as a specific civic health problem rather than a potential global biohazard. Those who drew attention to the permeability of laboratory boundaries allowed the debate over rDNA’s safety to leave the realm of science and enter local politics. From the outset, the inclusion of biological alongside physical containment criteria reflected an admission that the boundaries of the laboratory were not absolute (Wright 1994, p. 169). The left-wing scientific group Science for the People (SfP), which had emerged from the antiwar protests of the late 1960s, advanced this critique when its members in Cambridge became concerned with recombinant DNA. The Boston-Cambridge chapter of SfP was not represented at Asilomar in 1975 but argued in an open letter to the participants that rDNA was not only a problem for research scientists but for a “larger community” that was touched by the operations of laboratories. “Decisions at this crossroad of biological research cannot be made without public participation,” the letter asserted.^5^ Their approach to rDNA in this manner continued. In the spring of 1976, the MIT chapter of SfP surveyed 150 laboratory technicians, graduate students, and others, drawing attention to the intersection of biosafety concerns with other issues such as the lack of clean places to eat, insufficient worker training, the absence of occupational health services, and the need for a system of safety practice enforcement.^6^

The framing of rDNA as a potential community hazard by SfP set the conditions for a dramatic upwelling of concern in Cambridge that brought the carefully wrought consensus of Asiomar into new political terrain. Following the release of draft NIH guidelines for rDNA research in December of 1975, Harvard published brief notice of a public meeting in the classifieds section of the February 19, 1976 issue of the *Cambridge Chronicle* to discuss its intentions to apply for a grant to “renovate” space inside its Biological Laboratories for research on “animal cells, tumors, viruses, and plasmids.” The use of rDNA was not explicitly mentioned (Anon. 1976). This sparsely attended meeting, required as part of the permitting process for construction in Cambridge, did not capture the debate simmering within Harvard over what was intended to be a P3 facility for rDNA research. Several Harvard biology professors, some aligned with SfP, objected to the facility on safety grounds—Carroll Williams said that he would move his offices if the facility were built, and Ruth Hubbard asked about the danger of visitors to the lab becoming infected (Gottlieb and Jerome 1976, pp. 9, 29). Harvard’s internal debate drew outside notice when construction for the laboratory was reviewed by Harvard’s Committee for Research Policy, a body established during the Vietnam War to evaluate controversial research proposals. Scientists worried about the safety of rDNA research invited Cambridge councilperson Barbara Ackermann to observe. More attention soon followed, resulting in a June 8, 1976, cover story on “Biohazards at Harvard” in the *Boston Phoenix* (Gottlieb and Jerome 1976, p. 36; Mendelsohn 1984).

This article came to the desk of Cambridge Mayor Alfred Vellucci after one of his secretaries noticed it.^7^ Although Vellucci was unfamiliar with molecular biology, he was well versed in Cambridge’s town-gown politics. As a councilperson and mayor, he had regularly sought confrontation with Harvard, which served the dual purpose of burnishing his populist credentials and advancing the interests of the city, whose residents were wrestling with the loss of manufacturing jobs. Vellucci later explained that the universities were “big” and that “there should be constant pressure on them […] because they can be very helpful” (Stahl 1990, p. 1). Vellucci used the city’s jurisdiction over seemingly peripheral aspects of academic operations, such as zoning and construction, to make theatrical points regarding university expansion that served, to his mind, pragmatic ends. Vexed by the parking woes caused by commuters to Harvard, he proposed paving over Harvard Yard, yielding the double benefit of prompting Harvard to improve bus transportation and pressuring MIT to build more garages for its commuters.^8^ In another notorious episode, Vellucci suggested that Harvard would keep alligators in a moat during discussions of the final design of a new building for the School of Education (Kann 1963; Stahl 1990).

Read through Vellucci’s eyes, the *Boston Phoenix* article contained provocative observations about both the nature of the hazards posed by rDNA and the decision-making process that brought rDNA to Cambridge. Daniel Branton, the chair of Harvard’s Committee on Biohazards, had attempted to deflate questions about the safety of Cambridge residents with an appeal to the general risk of rDNA that seemed to imply widespread community risk. “Why talk just about Cambridge? [...] There are people from all over who come to Harvard every day […] if an organism escapes we might as well be talking about the world” (Gottlieb and Jerome 1976, p. 28). Yet as the P3 laboratory’s construction process proceeded through modestly publicized and sparsely attended meetings it appeared as if its fate would be decided with little public input. When asked, Cambridge’s city manager insisted there was no need for municipal involvement because there was “no health issue that has been raised about the facility” (Gottlieb and Jerome 1976, p. 28). 

Vellucci saw rDNA as a resonant issue. In densely populated Cambridge, work was never far from home and workplace hazards could easily become municipal hazards. Residents of industrial East Cambridge, which formed Vellucci’s political base, had long been frustrated that decisions about the placement of worksites were made without their health in mind (Kiechle 2017, pp. 198–232; Schlichting and Kiechle 2020). Vellucci strongly suspected that his own father had died from a disease contracted after years of working in Cambridge.^9^ SfP added to this concern, warning that the exhaust system for the proposed laboratory would vent in the direction of a daycare center.^10^

Vellucci convened hearings on the proposed laboratory that soon encompassed the suitability of rDNA research in Cambridge. He asked, “would recombinant DNA experiments be safer if they were done in a maximum-security lab, a P4 lab, in an isolated, non-populated part of the country? Would this be safer than using a P3 lab in one of the most densely populated cities in the nation?”^11^ During the hearings, Vellucci turned to the sewer system as the most cogent illustration of the connections among research and the city’s residents. He rebutted assurances of rDNA’s safety from a Harvard biochemist with the recollection that “when I was a little boy I used to fish in the Charles River, and I woke up one morning and found millions of fish dead in the Charles River, and you tonight tell me that you’ve dumped chemicals into the sewer system and the sewer system overflows into the Charles River.”^12^

Within the rhetorical pyrotechnics of the hearings, Vellucci’s enduring contribution to the legacy of Asilomar was in reinforcing the framing rDNA as a matter of municipal public health. In 1976, rDNA had been contemplated as a general, potentially existential, danger, or as an occupational health issue, but not yet as a public health problem. Vellucci and others on the City Council emphasized the local nature of the potential risks borne by communities near rDNA sites. He pointedly asked the Dean of the Harvard School of Public Health, “if it happened in [your hometown of] Brookline how would you feel?” The Dean conceded, “I would be sympathetic with the position you’ve taken.”^13^ This threat suggested a new pathway for regulating rDNA. Cities had longstanding and broad police powers to act in matters of public health, to “remove or prevent” any activity that might be judged “nuisances, sources of filth and causes of sickness within its town [or] may be injurious to the public health” (Massachusetts Association of Health Boards 1995, sections 3–1 and 3–2). If Cambridge decided rDNA was hazardous, the city could assert the authority to prohibit the technique, much to the chagrin of biologists at Harvard and MIT.

The Council took two steps based on its public health authority in the summer of 1976. It established a three-month moratorium on all rDNA work in the city and then created the Cambridge Experimental Review Board (CERB) to gauge the “hazards of experimentation to the citizens of Cambridge,” specifically, its potential “adverse effects on public health.”^14^ The membership of the CERB drew from a wide range of the city’s denizens: clerical workers, physicians, an ex-Mayor, neighborhood organization leaders, and the SfP-aligned Tufts sociologist and future rDNA chronicler Sheldon Krimsky (Krimsky 1978). The CERB took the novel form of a “citizen[s’] court” as they grappled with what standards to set for rDNA research; it needed to be proven safe for the city “beyond all reasonable doubt.”^15^

The CERB stated in its January 1977 report that the “philosophical issues” raised by genetic engineering were “beyond the scope of our charge.” However, decisions weighing the “risks and benefits” of rDNA research should not be resolved within “the inner circles of the scientific establishment.”^16^ Its broad membership base acted as a counterweight to the interests of scientists in advancing their research, and “insured that the ‘empathy factor’[…] the concern that the institutions proposing the research would lose valuable funds or that qualified researchers would leave in the event of a ban on the research, was never an issue in the deliberations.”^17^ Considering the uncertain nature of rDNA research’s hazards, the CERB went beyond the recommendations of the NIH guidelines, making a “concerted effort to assess the risks” of P3 or P4 level research to the Cambridge community, and recommending additional restrictions on research and health monitoring of researchers in rDNA facilities.^18^

The CERB’s recommendations were adopted by the Council as modifications to the city’s public health ordinances. The rDNA ordinance prohibited research requiring the highest level of containment, P4, and required “strict conformity” with the existing NIH guidelines. It established the Cambridge Biohazards Committee (CBC) to oversee the institutional biohazard committees required by the NIH.^19^ The fact that the CBC dovetailed with the institutional oversight of rDNA required by the NIH has retrospectively been framed as a success for scientific autonomy (Lipson 2003, p. 8). Scientists in Cambridge did not share this view at the time. After the Cambridge rDNA ordinance was passed, the Nobel Prize-winning MIT molecular virologist David Baltimore wrote to the MIT provost emphasizing that there was research he wanted to conduct that was “legal by NIH” standards but forbidden by the Cambridge ordinance. “We need to be cloning now.”^20^ Baltimore was lucky in that MIT already had a P3 laboratory; until construction was complete in the Biological Laboratories, Harvard biologists working with rDNA had to seek bench space at MIT, Cold Spring Harbor Laboratory in New York, or even further afield.^21^

Writing as the ordinance came into effect, Melvin Chalfen, the new Cambridge Public Health Commissioner, acknowledged that “no other locality in the country (or the world) as far as I know has such prohibitions.” Chalfen conceded that many biologists in Cambridge “feel they are severely handicapped in their work both in performance and in their position and progress vis a vis their colleague[s] in localities outside of Cambridge.” However, he was not sympathetic to the complaints that regulations were too onerous. If appropriately safe organisms to perform work with did not exist, it seemed like “a motivation to create them.”^22^

Unlike national deliberations, scientists possessed relatively little power to sway the course of public health regulations once they were set. Indeed, local power over rDNA in Massachusetts only grew. Initially, the bounds of municipal authority were uncertain enough that several commercial firms approached MIT’s Institutional Biohazards Committee to review their proposals prior to seeking public approval.^23^ City authorities in Boston and Somerville who later wanted to regulate rDNA were also unsure if they had the legal basis to do so.^24^ Ironically, it was the choice of more confrontational tactics in the mid-1980s by a prominent Cambridge chemical engineering consulting firm, Arthur D. Little, that shored up the foundation of rDNA regulations. The firm sued to challenge another municipal prohibition on the transfer and storage of toxic chemicals that it had been contracted to study by the U.S. Department of Defense. The Supreme Judicial Court of Massachusetts upheld the prohibition, finding that federal legislation did not preempt more restrictive local health regulations, and that, moreover, the ultimate authority to weigh the risks of research that might result in harm to the inhabitants of a town rested with its public health authorities, which were not “obliged to provide a statement of the reasons which support its adoption of a regulation.”^25^

## Commercial Biotechnology and Asilomar: From Evasion to Accommodation

In the early 1980s, the emergence of the modern commercial biotechnology industry shifted the conversation around rDNA. While rDNA had first been seen as a tool for research, biotechnology and pharmaceutical firms saw it as novel means of producing valuable therapeutic compounds. As a result, commercial uses of rDNA soon became very prominent. New biotechnology firms using rDNA proliferated, especially in the San Francisco Bay Area and the GBA. Historians of biology have generally assumed that the impact of Asilomar-inspired regulations on this emerging industry was limited. Companies were not federally funded, so they were not subject to regulation through NIH grantmaking policies. The most significant regulatory moment was the “non-decision” of Congress to regulate private rDNA (Wright 1994, p. 11). Following firms in the San Francisco Bay Area, historians have gained the impression that mobility of biotechnology start-ups and their distaste for regulation allowed them to evade local governance by moving to more welcoming communities nearby, creating a deregulatory race to the bottom (Hughes 2011; Yi 2015). However, tracing the actors involved in the promulgation of the so-called second wave of local rDNA regulations during the early 1980s shows that firms soon found benefit in accommodating local regulations rather than avoiding them (Krimsky 1982b). The location choices of two major early firms in Cambridge, Biogen and the Genetics Institute, suggest that there were equal incentives, rooted in a firm’s operations and infrastructure, to reach accommodations with local regulation.^26^

As the *New York Times* reported in 1982, biotechnology executives publicly decried local regulation. “The purpose of this industry is not served by Draconian regulatory measures,” intoned an attorney for California-based Genentech. The *Times* attributed the early rapid growth of the industry in California to its less “stringent regulations than those in Massachusetts towns” (Radding 1982). Biotechnology companies did seem to have every incentive to avoid regulations. Despite the notice attracted by a handful of initial successful public stock offerings, most biotechnology companies struggled to define their value to investors—when products were a decade or more in the future there were few ways to know if a company was just a concept or a credible investment. The scientific productivity of a firm stood in as an important proxy for its future value (Fox 1982; Willoughby and Blakely 1989, p. 9). Any obstacle to starting research was a significant consideration.

Unlike universities, biotechnology firms could move to avoid local regulation. Studies of biotechnology firms in California suggested that they favored municipalities which adopted a lenient or indifferent approach to rDNA (Rea and Scotchmer 1988, p. 33). The general counsel of Genentech recalled that the firm sought space in industrial South San Francisco because they were concerned about further public demands for regulation in other communities: “people were marching in Berkeley chanting ‘we shall not be cloned.’”^27^ Also anticipating regulation, Berkeley-based Cetus established its recombinant DNA laboratories in neighboring Emeryville. When Emeryville passed its own local regulations, Cetus remained, hoping that the rules would not be enforced (Krimsky et al. 1982, p. 15).

Firms in the GBA reluctantly, but ultimately, chose to embrace regulation and the stability it offered. Biogen’s decisions as it became the first commercial biotechnology firm to choose a location in Cambridge in 1980 demonstrate how the operational needs of a biotechnology firm could draw it to zones of stricter but stable regulation. Initially, Biogen embraced evasion on a global scale. Biogen emerged from an international meeting of scientists in Geneva in 1978, including several from Harvard and MIT. Although it established its headquarters in Switzerland, Biogen structured itself as an international constellation of laboratories with the aim of shielding the company from the whims of any one city or nation.^28^ Fearing that Cambridge’s regulations would delay his laboratory’s research, Harvard chemist Walter Gilbert, one of Biogen’s founding members, used his connections through the firm to conduct pivotal gene cloning experiments in the United Kingdom’s Porton Down biological warfare research laboratories (Hall 1987, pp. 249–265). When Biogen decided to establish a U.S. laboratory in January of 1980, one of its investors warned that “activities in Cambridge would be limited by the local biohazard committee,” and suggested several alternatives in neighboring cities.^29^ Like California biotechnology firms, Biogen stressed its locational flexibility: writing in its letter of inquiry to the CBC that “at this stage it is probably worth underlining that our choice for Cambridge is only a preference, and certainly not the only location envisageable [sic] in the United States.”^30^

However, Biogen came to see Cambridge as highly desirable, if not necessary, for its prospects. Internal Biogen documents reveal that American members of Biogen’s Scientific Advisory Board urged their European colleagues that “vicinity to MIT or Harvard is of key importance for the performance of the lab.” By dint of the GBA’s geography, this “for all intents and purposes, requires a Cambridge location.” Biogen’s Swiss Vice President of Finance and Administration explained from Geneva that that although “Cambridge has established [rDNA regulations …] that are more stringent than elsewhere in the USA” he had found no suitable sites in neighboring Somerville, and in another city, Malden, “the authorities [… also] raised concern about the type of activity envisaged.” The only plausible “fallback” was a 10-minute drive away. Ultimately, Cambridge was preferable because “the battle […] has been fought [… and] there is uncertainty in surrounding communities.”^31^

Biogen’s interest in stable regulations was so deep that it actively negotiated to revive the 1977 Cambridge rDNA ordinance, which was in a weak state by 1980. Having established oversight over Harvard and MIT, which were also bound by the NIH guidelines through their funding sources, the CBC was well on its way to becoming dormant in 1980. In the spring of 1979, the chair of the CBC reported that due to loss of members there would be “no duly constituted committee” capable of enforcing regulations.^32^ Biogen’s letter of inquiry revealed that a 1978 ordinance had inadvertently repealed the rDNA regulations passed in 1977! 33 James Sullivan, the City Manager, convened the CBC to review Biogen’s proposal, remarking that biotechnology in Cambridge would be “prominent and prosperous.” The charge of the revived CBC was “not to obstruct such activity,” but to “establish guidelines” in conversation with Biogen (Elliot 1980).

Biogen worked closely with the CBC to then modify the ordinance with an eye towards commercial biomanufacturing as well as research. When written in 1977, the Cambridge ordinance had focused on research uses of rDNA rather than the “large scale” uses of industrial biotechnology. Academic research with rDNA generally took place using culture volumes of less than 10 liters, but fermentation for producing therapeutic compounds on a pilot plant scale required cultures at least ten times larger in magnitude, and mass production at the scale of the pharmaceutical industry involved volumes in the thousands of liters, raising new questions issues of waste disposal.^34^

Biogen faced skepticism at its hearings. Vellucci asked if allowing Biogen to work “within a three minutes’ walk from the MIT laboratories” was worth the possible safety risks. “What if they spill some of the stuff on the floor? [...] They have no way of dumping their waste. Will they dump it in the sewers of Cambridge?” (McLaughlin 1980). Krimsky, a member of SfP, warned that no federal regulations restrained the use of rDNA by commercial entities and recommended that biotechnology firms pay for state-certified consultants to monitor wastewater to ensure that no organisms drifted into the sewers. A former member of the CERB spoke as “Mrs. Average Citizen.” She and her neighbors in East Cambridge, abutting the Biogen site, were “frightened, we are very, very frightened […] Once again industry is coming to an uninformed population and proposing to put in a recombinant DNA factory” (Sege 1980a).

Despite these public critiques, Biogen was anxious to reach an understanding with the city. In December, the firm’s British president met with a reconvened CERB to discuss how the revised ordinance might apply to the firm’s operations. The CERB’s inquiry revealed troubling questions—many homes and businesses around Biogen’s site were serviced by wooden sewer pipes over 100 years old, notorious for leaking into basements. No one could yet say if Biogen’s laboratories would be connected to concrete pipes or these wooden pipes, raising the prospect that, with time, organisms from the laboratory’s effluent would make it into the homes of East Cambridge. Biogen affirmed that it would sterilize its waste on-site before passing it to the city sewer system, an additional step that was still far more affordable than hauling it away.^35^

During these discussions Biogen, unlike West Coast firms, was more concerned with resolving the questions of obtaining permits “expeditiously” and definitively rather than on the most lenient terms (Sege 1980b). Biogen accepted that the permitting process for large-scale fermentation would include health monitoring, inspections, and even fees to support this work. However, Biogen insisted that the approval process itself should be a one-time occurrence. The prospect of “annual permit reviews” by the city threatened that at any given point “the rug could be pulled out from underneath” Biogen’s facilities—a daunting prospect for a company preparing to spend millions of dollars on the site.^36^ The Council passed a revised rDNA ordinance in April of 1981 by an eight-one vote, with Vellucci dissenting. Biogen’s president commented that the ordinance held “no surprises” for the company, because its provisions included steps it had discussed with the CERB, such as a separate process for large-scale rDNA work and a mandate for companies to bear the cost of complying with the occupational health and environmental safety provisions of the ordinance. An annual inspection replaced a full-scale public permit review. Biogen proceeded with construction (Anon. 1981).

The journey of the Genetics Institute through the GBA underscores why biotechnology firms came to favor known regulations over evasion. Just as Biogen started to examine options for a North American laboratory, Harvard stirred a different kind of controversy when it proposed directly investing in a biotechnology firm to be founded by the Chair of its Department of Biochemistry and Molecular Biology, Mark Ptashne (Etzkowitz 1983, pp. 199–200). Although this scheme fell through, Ptashne founded the Genetics Institute with private backing. Like Biogen, the scientist-founders of the Genetics Institute favored staying close to their laboratories, ideally within 15 minutes travel of Harvard by public transportation.^37^

In late 1980, the Genetics Institute started to plan for a laboratory in a former silversmithing building near Harvard in the neighboring city of Somerville. Somerville had not passed a recombinant DNA ordinance after Asilomar because the city did not host academic rDNA work. In a letter to Somerville declaring its intentions, the Genetics Institute insisted that early concerns for the safety of recombinant DNA were illusory, the work of “left wing radicals who wanted to exploit the issues for Marxist reasons and conservative politicians who wanted for unknown and selfish reasons to hit the headlines” (Sege 1981). Ptashne, who had sparred with Vellucci during the Cambridge rDNA hearings, proclaimed that he “would be willing to swallow anything we make in the lab” (Davidson 1981a).

The 200 residents of Somerville who gathered in early 1981 to consider the laboratory were unmoved by these assurances. “You don’t have to be a Nobel Prize winner to know that accidents happen,” commented one participant (Somerville Officials Debate DNA Law 1981). Somerville’s Sixth Ward Alderman invoked Asilomar when he asked the scientists “if five years ago this was frightening to people of your background, how do you think it feels to people in Somerville?” (Davidson 1981b). Not only was Somerville’s Board of Aldermen unwilling to issue a permit to the Genetics Institute, but the city also appeared ready to consider prohibiting rDNA research entirely. Unwilling to wait for the resolution of these issues, the Genetics Institute ceased consideration of the site (Connolly 1981).

The Genetics Institute then turned its attention to Boston’s Longwood Medical Research Complex, which contained the laboratories of Harvard Medical School. Boston also had not issued regulations at the peak of public and scientific concern for the safety of rDNA. The Institute hoped to quickly strike a deal with Brigham Women’s Hospital to lease laboratory space in a former rehabilitation facility. However, residents of the working-class Mission Hill neighborhood that adjoined the hospital to the south were primed and organized to question the safety of biomedical research. The neighborhood had spent the better part of the previous decade sparring with Longwood-area hospitals and laboratories regarding the negative environmental impacts of emissions from the proposed Medical Area Total Energy Plant (MATEP), a diesel-burning power generation station to be placed in Mission Hill. The Genetics Institute soon found flyers across the neighborhood asking “MATEP and DNA: More of the Same?” (Waitzkin 1977; Grady 1977; Waitzkin and Sharratt 1977; Goldberg 1981, pp. 88–89; Krimsky et al. 1982, pp. 7–10).

Boston City Councilor Raymond Flynn, a populist with a history of focusing on the impact of citywide decisions on the neighborhoods of his constituents, which had previously included opposing busing to integrate schools, soon introduced an rDNA ordinance (Dreier 1993). The preamble warned that “rapid advances in recombinant DNA technology have far surpassed the ability of government to enact legislative safeguards to protect public health and safety,” highlighted by the absence of “effective enforcement procedures” for “commercial ventures involving DNA research.” These issues were acute in the Mission Hill neighborhood, “a heavily populated residential community” shared with “large numbers of hospital patients who are further ‘at risk’ in cases of accidents involving DNA research.”^38^

When Flynn held hearings in Mission Hill, hundreds of residents appeared to discuss the matter, questioning the motives of “venture capital bandits” and the risks of research to the community. Attempting to moot the issue, Brigham Women’s Hospital promised that it would require adherence to the NIH rDNA guidelines as part of its lease agreement, but the newly formed Mission Hill DNA Committee pressured Boston to monitor the sewers of all laboratories carrying out rDNA work in the city and prohibit commercial rDNA firms until they fully complied with the ordinance, a potentially costly delay.^39^ This suggestion drew intense counter-lobbying from Boston’s biomedical research community, and the final regulations more closely resembled Cambridge’s 1981 ordinance (Roberts 1981; Krimsky et al. 1982, pp. 7–10). Genetics Institute, while it was able to open its laboratory in Boston, had been dealt yet another sharp reminder of the importance of “the issue of municipal regulation,” in the recollection of its incoming CEO, Gabriel Schmergel. Because its Boston location could not support expansion into manufacturing, he endorsed relocating to Cambridge, where the Genetics Institute settled in 1982.^40^

As individual companies came to accommodate local regulation, so too did Cambridge’s leaders start to embrace biotechnology. While rDNA first entered city politics as a threat to public health, it soon became a potential boon to economic health. After the Second World War, Cambridge and other manufacturing cities in Massachusetts watched deindustrialization hollow out job rolls and tax bases. Cities tried many remedies for this decline, from tax credits to redevelopment, in vain. Indeed, the other hazard associated with technology that Vellucci cited during the 1976 Council hearings on rDNA was not epidemiological but economic. In the early 1960s Cambridge seized dozens of acres of East Cambridge, razing homes and businesses, for a site intended to draw a NASA headquarters that was never built (Winling 2017, pp. 156–159). He recalled, “I sat in that seat […] fighting the coming of the NASA site in Cambridge. And I predicted that the whole thing would collapse and it collapsed.”^41^ These challenges were compounded by the 1980 passage of Proposition 2 ½ in Massachusetts, part of a national anti-tax movement. It limited taxes on residential property and gave cities across the Commonwealth new incentives to encourage tax revenue-generating commercial development (Geismer 2015, pp. 259–267).

The empty NASA site sat across the street from Biogen’s 1980 laboratory site, offering a prime location for its pilot biomanufacturing plant. When Biogen sought permission to expand its operations in 1982, it focused on promises that the company would bring life to the “empty” districts of the city and employment to builders and service workers from the facility.^42^ In a striking shift from 1976 or 1980, no citizens appeared at the hearings to contest Biogen’s application. “I think that’s the biggest news” commented the chair of the CBC (Anon. 1982). Promises of jobs and tax revenue converted even the most stringent critics. The CEO of the Genetics Institute recalled that in a private meeting before a vote to approve the company’s new site in West Cambridge in 1982, Vellucci explained that he was interested in the possible tax revenue and employment that the Genetics Institute would bring to Cambridge, but for political purposes during the public hearing, “I’m going to roast you [...] but don’t worry I want you here.”^43^ Vellucci was open regarding his change of heart at a ribbon-cutting ceremony for Biogen’s new pilot production plant a year later. He joked that the firm’s treasurer was headed to city offices with the tax payments as he spoke (Keough 1983).

## Regulating the Risk of Risk

If the fears raised by Asilomar were ameliorated by time, experience, and interest in the economic benefits of biotechnology, one might expect the frequency of local regulations to diminish. However, in Massachusetts the number of local rules governing rDNA research only increased after 1982. This third and accelerating wave of regulations in the 1980s, 1990s, and beyond reflected a new development—cities attempting to encourage economic development through public health. Public health regulations modeled on Cambridge’s ordinance became a site of ongoing lobbying and intervention not by those concerned for the safety of rDNA but by those concerned for the cultivation of the biotechnology industry by strengthening its financial infrastructure.

Although it produced fewer dramatic incidents than the 1970s or early 1980s, concerns about public reactions to rDNA remained a constant presence during the biotechnology industry’s growth. Despite earning the dismissive appellation of so-called killer tomato syndrome, biotechnology advocates were worried that a deep reservoir of public distrust remained from Asilomar that could well up at any moment (Corliss 1990). Biotechnology companies feared that this distrust would deprive them of access to the civic infrastructure at critical moments. In one episode, a fire alarm at Biogen was (inadvertently) triggered, but the Cambridge Fire Department would not enter the building for fear of what the laboratories contained.^44^ New industry groups, such as the Massachusetts Biotechnology Council (MBC), came to embrace the stability of local regulations as a hedge against shifts in public opinion. Although founded in 1984 by a group of eleven companies to lobby the federal government, the MBC soon became deeply involved in local politics.^45^

The MBC warned that a patchwork of local regulations could create an “onerous” permitting environment, especially when a locality only started to consider what regulations to adopt when approached by a biotechnology firm (Feinstein 1990, pp. 13–14). In addition to public health, each Massachusetts community had its own regulations on land use, and changing these zoning regulations could trigger a further and potentially contentious process of local approval. This point had preoccupied the CERB when Biogen applied to establish operations in Kendall Square. The board only belatedly realized that it had to consider land use regulations when a member who also sat on the Cambridge Industrial Development Finance Agency, which helped arrange a $4.5 million construction loan for Biogen, inquired if its preferred site was zoned for rDNA work. This question would shadow other companies given that NIH guidelines made no mention of the suitable “external” environment for rDNA research—only its “internal” safety arrangements.^46^ Surveyed in 1990, two-thirds of companies were concerned that local regulations would be an obstacle, and preferred regulatory schemes that were “smooth” and “without harassment” from undue public concern (Feinstein 1990, pp. 17, 28).

In light of these concerns, the MBC and other advocates of the biotechnology industry reframed the essence of the Cambridge regulations, stressing not that they were based on a misguided assumption of danger but that they were consistent, transparent, and reliable. A report commissioned by the MBC and other Massachusetts economic development agencies praised Cambridge for the predictability of its regulations. Companies in Cambridge had confidence that “these rules were set and would not change precipitously” (Feinstein 1990, p. 17). While Cambridge’s regulations might have been more onerous, they were set. David Glass, president of the MBC, cautioned that discussion too frequently turned to “how to adopt a regulatory scheme rather than adopt a *stable* regulatory environment” (Corliss 1990).

In the early 1990s, as city leaders in the GBA became interested in the economic benefits of biotechnology companies, the MBC sought to foster local regulations on terms favorable to the industry. In its outreach efforts, the MBC stressed that the safe, “modern, clean, and attractive work environments” of biotechnology laboratories made them suitable neighbors anywhere.^47^ The MBC suggested that a community hoping to welcome biotechnology should establish municipal codes based on the NIH guidelines as a means of “serv[ing] and protect[ing] the community while creating an atmosphere which encourages biotechnology companies to locate in a community.”^48^ The model ordinance it offered replicated many elements of Cambridge’s, placing power to approve and monitor rDNA work in the hands of the city or town’s board of health, rather than public hearings, on terms favorable to a prospective biotechnology firm.^49^

While an observer of the biotechnology industry in the early 1980s would have heard a litany of critiques of local intrusion, the MBC shows that historians of biotechnology should consider if there may have been subsequent reasons for the industry to endorse regulations at the local level. The appeal of regulation to the MBC reflected the systemic challenges that the industry faced securing financing for laboratory construction. After biotechnology firms abandoned the hope of conducting work in university laboratories, they bore the full costs of constructing laboratories. These spaces could cost four times as much as standard office space and were difficult to retrofit for any other use (Hower 1992). The construction of these facilities depended not on speculative venture capital so often associated with biotechnology, but on construction loans from conventional banks. Despite the importance of credit markets to fashioning the built environment and public life of modern cities (Jenkins 2021), financing for the physical spaces of science has not often been discussed as part of the economic history of science. Compared to the inconvenience of regulation, the threat of not having access to credit was far more worrisome as the industry sought credit to expand.

Business histories of biotechnology firms have focused on their efforts to attract funding from speculative investors and the research strategies they subsequently pursued (Kenney 1986; Pisano 2006; Rasmussen 2014), creating a species of what later commentators called fast-paced “venture science” (Sunder Rajan 2006, p. 136). As Biogen’s president explained to the CERB: “What is Biogen selling? Brains, really, at this stage of the game.”^50^ However, although a new firm might be successful in raising funds from investors to fund research, this did not correspond to gaining support to build the laboratories in which research could occur. Venture investors did not want to pay for “bricks and mortar,” they wanted their investment to finance “only scientists” as companies sprinted to claim credit for new discoveries.^51^

Yet the creation and approval of physical buildings was a vital step toward satisfying investors’ expectations. In December of 1985, the MIT-affiliated firm Repligen, the second private biotechnology company to seek a permit in Cambridge, appeared before the CBC to present a slideshow demonstrating the containment features of its new laboratories it leased at One Kendall Square, a short walk from the MIT campus. This was not a conventional real estate development. Its leader, David Clem, had mortgaged his home to partially finance the project and was willing to act as a “venture capital” real estate investor– taking far riskier tenants in the life science and computing sectors than other landlords in exchange for equity (Scheffler 2023). The treatment area in the basement was “essentially a concrete bunker with a thirty-one-inch-high dam in the doorway,” sealed with caulk and waterproof paint. The facility, Repligen’s representative urged, was on a “fast track” construction schedule to meet its obligations, laid out in its business plan, to have a functional laboratory by January 23, 1986. He asked that Cambridge review and certify the facility before the date. The CBC just barely accommodated his wish, scheduling the final inspection for January 21.^52^

Firms in other sectors would have dealt with the expenses of creating new research and production spaces through the process of amortization —obtaining loans that allowed them to spread the large one-time expense of construction over many years. However, biotechnology companies faced a chronic mismatch between their financial health as understood by speculative investors versus how lenders assessed their health from balance sheets. Research as companies worked to bring products to market still counted as a loss, and bankers did not place a value on the promise of future discoveries. “It’s easy to be fooled by people in white coats,” quipped one banker. Bank regulators grappled with “conceptualizing how to protect their security interests in intangible property,” rather than “bricks and mortar” (Welch 1984).

Challenges financing construction did not abate even as the biotechnology industry became more familiar to bankers. In the wake of the mid-1980s Savings and Loan crisis, lenders were very concerned about issuing adequately secured loans. “The creditworthiness template that the banks are using doesn't fit biotech," warned a Cambridge- based biotechnology consultant (Hower 1992). “Banks will lend money to a pizzeria that has positive cash flow,” complained Richard Aldrich, CFO of Cambridge-based Vertex, of his own firm’s difficulties finding construction financing (Rosenberg 1992).

Businesses investing in biotechnology real estate treated local responses to biotechnology as a systemic element of risk to their investors.^53^ Within the time horizon of investment in biomanufacturing sites, backers were anxious that no future objections would arise. “When the biotechnology industry decides to build a new manufacturing site […] they have to make a judgment as to how the taxation and regulatory scene will be that far into the future. Because Massachusetts has been so volatile in that area the biotech industry is afraid of the unknown,” commented Peter Bates of the Boston law firm Goodwin, Procter & Hoar (Lemire 1991). It was a commonplace observation among those financing biotechnology developments, especially large-scale biomanufacturing, that “public opposition can be a substantial risk to the development schedule of a new facility.” The “perception of biological hazard[s]” could seriously “delay regulatory approvals or impact site selection.” Thus understanding the possibility of these risks was one of the features of the “due diligence” carried out by financial institutions offering credit to biotechnology and biomanufacturing (Keller and Plath 1999, p. 645).

For these lenders and biotechnology industry advocates, Asilomar lived on as an element of the risk calculus that determined the availability of credit to the industry due to the fact that Cambridge and other GBA cities wrote the NIH guidelines into their local regulations. Long after public discussion quieted, financial institutions underwriting biotechnology firms or landlords considering leases approached the CBC for documentation that a firm had gone through the municipal regulatory approval process (Chalfen 1990, p. 160). Whereas the first surveys of local rDNA ordinances had been carried out by neighborhood activists concerned for community participation in decision-making, later compilations were carried out by groups either seeking to invest in or promote the biotechnology industry (Krimsky et al. 1982; Bernard et al. 1995, p. 6). As with other instances of research governance in 20th century biology, regulations that at first glance seemed to impede work ultimately served to shield it by easing financial risk or legal liability (Stark 2012, p. 77).

The appetite for stable locations grew with the involvement of the pharmaceutical industry in the GBA biotechnology cluster and its movement from research to biomanufacturing, which required additional civic infrastructure and approval. In 2009, the MBC unveiled its “Bio-Ready” campaign, surveying, and lobbying communities to preemptively ease the path for biotechnology development by ranking their regulations from “bronze” to “platinum.” Peter Abair, the MBC Director of Economic Development, proposed the effort not only to make financing easier for individual biotechnology firms but to make financing for biotechnology real-estate investment easier and more predictable across Massachusetts’s 351 communities.^54^ This 2009 MBC campaign emphasized the importance of zoning, sewer access, and prior approval for development in its ranking tiers, but the clearest pathway to a “platinum” rating was passing an ordinance modeled on Cambridge (Weisman 2009).

The MBC’s efforts reflected the ongoing potential for the safety concerns broached at Asilomar decades before to impinge on the industry’s operations. Major biotechnology developments in Massachusetts continue to spark local protest regarding the threat they might pose to host communities—a 2003 attempt by Boston University to construct a biohazards research facility in the South End faced was delayed by several years by neighboring communities concerned about the possible impact of spills or releases from the laboratory (Agyeman and Ross 2007), while more recent opposition to plans by the city of Revere to convert a former horse-racing track to laboratory space in 2022 were opposed by those who invoked the lab-leak theories associated with the origins of the COVID-19 pandemic (among other reasons).^55^ Biotechnology companies still establish facilities only to find that they selected a property not zoned for their work (Ryan 2023). While historians of biology might be drawn to evaluate *if* risks exist in these situations, from the perspective of biotechnology developers the very occurrence of these questions constitute a significant potential constraint on the industry. And the force of that constraint was (and still is) magnified by the financial mechanisms that underwrite the creation of laboratories and biomanufacturing facilities.

## Conclusion

The arrival of the fiftieth anniversary of the Asilomar conference is an opportunity to reflect on how historians of biology assign meaning and significance to events, a process of reflection which gains importance given the fact that Asilomar and the debates that followed in Cambridge are still invoked to provide lessons for the governance of new biological inventions (Parthasarathy 2015; Botelho 2021). In this process of reflection, the scalar and temporal boundaries placed around events help determine their meaning, but historians of biology have sometimes taken for granted the basis on which these boundaries are set. In the case of Asilomar, I have shown that following the development and expansion of commercial rDNA activity by the biotechnology industry produces a different understanding of its legacy than that which emerges from focusing on debates over the regulation of academic rDNA research alone. Whereas academics chafed at external regulation, especially by municipal authorities with no technical expertise, the financial concerns of biotechnology firms and their backers drew them to embrace the predictability that external, local regulations offered. These different views of regulation account for what appears to be a paradox in the history of rDNA in the GBA: even as concerns over its hazards receded, the number of local public health laws governing it proliferated. Rather than marking a challenge to the practice of science, regulations modeled on what began at Asilomar became part of the toolkit of community economic development.

While there is a deep tradition of scholarship on the material basis of laboratory research, there is less connecting the process of laboratory work with its immediate social and physical environment and contemplating the implications of these connections, which suggest that the social impact of science may be more local and immediate than often assumed. Even critical observers of biotechnology such as SfP, whose members and affiliates, such as Susan Wright and Sheldon Krimsky, turned their attention away from these local relationships. Although SfP had first insisted on the porous boundary between laboratories and the communities that surrounded them and played an important role in localizing debates over Asilomar, in the aftermath of the 1976 Cambridge debates and decline of scientific concern for the risks for rDNA these groups moved in the direction of considering the potential global impacts of biotechnology. While seeing a need to remain “diligent about the hazards of rDNA technology,” groups that emerged out of the SfP, such as the Coalition for Responsible Genetics, emphasized “broadening the explicit concerns of the Coalition to deal with [future] issues such as cloning, human genetic manipulation, and genetic screening programs.”^56^ These groups were largely absent from the later debates over Cambridge’s decision to host biotechnology firms and the changes it wrought on the city (Scheffler 2023, 2025, p. 207). Ironically, biotechnology firms remained more attentive to the embeddedness of their work in different communities than activists and historians of science following the activists. While the path followed by Asilomar in the GBA likely differs from other regions, the questions that it raises regarding the spaces and scales where emerging biotechnologies are regulated, and the communities to which these governance mechanisms are accountable, may be asked anywhere.

## Footnotes

1 A partial timeline of regulations compiled by the author is as follows: 1976: Cambridge; 1978: Amherst; 1981: Boston, Waltham, Somerville, Framingham; 1982: Newton; 1984: Worcester, Beverly; 1985: Lexington; 1986: Andover, Bedford; 1989: Methuen, Marlborough; 1990: Randolph; Grafton; 1992: Leominster; 1994: Burlington, Billerica. Current cities compiled by the Massachusetts Biotechnology Council (https://www.massbio.org/initiatives/bioready-communities, accessed June 2, 2024). Over the last twenty years the GBA has been a perennial contender for the largest biotechnology cluster in the world by different measures, and even when it slips in this ranking it remains the densest (Owen-Smith and Powell 2006; Philippidis 2024).

2 Measuring total rDNA use in the late 1970s and early 1980s is a challenging matter. Federal regulations applied to experiments using rDNA on a small scale, so a single large-scale use of rDNA for manufacturing (e.g. fermentation) would easily involve more rDNA modified bacteria. Since industrial uses were not regulated, there are no records of industrial use that would even allow this comparison (Wright 1980, p. 29). Accounts suggest, however, that commercial-industrial uses of rDNA predominated after 1982. A member of the NIH Recombinant DNA Advisory Committee, David Baltimore, retrospectively estimated that 95% of rDNA use was commercial by 1982 (Crotty 2001, p. 126).

3 There are antecedent echoes of this perspective in early laboratory ethnography, particularly the idea of the laboratory as a production site for papers that requires different energetic and labor inputs (Latour and Woolgar 1979, p. 52). Other historians of biology have examined the extended forms of infrastructure for modern biological research (Creager and Landecker 2009). These thoughts have been given methodological force by emphatic calls to make *maintenance* rather than *innovation* or *invention* an appropriate target for study by historians of technology (Ensmenger 2018; Vinsel 2021; Monserrate 2022).

4 My interpretation and timeline here is drawn from Susan Wright’s account. Walters LeRoy, a bioethicist at Georgetown University, joined the committee in September 1976. Membership data compiled by Wright (1994) in her Table 4.1. The types of physical and biological containment for experiments could thus be thought of as a matrix—each proposed experiment had both a recommended physical containment ranking and a set of recommended safe strains, see Wright’s table 4.3.

5 Open Letter to the Asilomar Conference on Hazards of Recombinant DNA, February 1975. Authors included Johnathan Beckwith, Jonathan King, Paolo Strigini, Luigi Gorini, Kostia Bergmann, Kaaren Janssen, and Annamaria Torriani. Digital copy in National Library of Medicine Profiles in Science, Maxine Singer Papers. http://resource.nlm.nih.gov/101584644X101 (Accessed June 23, 2024).

6 Biology Workers’ Health and Safety Committee Survey Summary, 11 May 1976. MIT Archives and Distinctive Collections, AC7, Box 31, Folder 12.

7 Several accounts suggest that Vellucci was first approached by concerned biologists at Harvard, but this is not corroborated by the oral history of the events produced a year afterward, which included Pat Centanni, the Mayor’s secretary. Alfred Vellucci Interview with Rae Goodell, 9 May 1977, MIT Archives and Distinctive Collections, MC-100, Box 14 (hereafter, Vellucci Interview), pp. 40, 44.

8 Vellucci Interview, p. 36.

9 A heart-lung condition contracted while at Swift and Company, a meatpacking plant that also manufactured cleansers. Vellucci Interview 1977, p. 2.

10 Boston Area Science for the People, “Are we Unsuspecting Research Subjects??????????,” flyer dated 23 June 1976. MIT Archives and Distinctive Collections, MC-100, Box 37, Folder 555.

11 Transcript of Cambridge City Council Hearings on rDNA, 23 June 1976, pp. 55–56. MIT Archives and Distinctive Collections, MC-0553, Box 50, Folder “CCC Hearings rDNA Transcript.” Hereafter, CCC rDNA.

12 CCC rDNA, pp. 71–73.

13 CCC rDNA, p. 221.

14 James Sullivan [City Manager] to Cambridge City Council 6 August 1976. Cambridge Public Library Cambridge Room, Cornelia Wheeler Papers, Box 5, “DNA Research.”

15 Cambridge Experimental Review Board [Mary Lucille Banach, Sheldon Krimsky, John Brush, William LeMessurier, Daniel Hayes [chair], Mary Nicoloro, Constance Hughes, Cornelia Wheeler] report to City Manager [James L. Sullivan] transmitted 5 January 1977, pp. 1–2. Cambridge Public Library Cambridge Room, Corneila Wheeler Papers, Box 5, “DNA Research” (hereafter, CERB Report).

16 CERB Report p. 1.

17 CERB Report, p.4.

18 CERB Report, p. 3.

19 City of Cambridge Ordinance #1895, “Ordinance for the Use of Recombinant Molecule Technology in the City of Cambridge” (27 January 1977); https://collections.nlm.nih.gov/catalog/nlm:nlmuid-101584644X124-doc

20 David Baltimore to Walter Rosenblith and Walter Milne, 14 June 1979. MIT Archives and Distinctive Collections, AC7, Box 24, Folder 25–correspondence cancer project 1973–1976 (1/3).

21 CBC Minutes 24 August 1978, p.1. Cambridge Public Health Department, Melvin Chalfen Papers [unprocessed], “Cambridge Biohazards Comm.”

22 Melvin Chalfen to Walter Rosenblith [MIT Provost], 21 June 1979. MIT Archives and Distinctive Collections, AC7, Box 24, Folder 25--correspondence cancer project 1973–1976 (1/3).

23 Minutes of the Cambridge Biohazards Safety Committee Meeting, 29 August 1979, p.2. Cambridge Public Health Department, Melvin Chalfen Papers [unprocessed], “Cambridge Biohazards Comm.”

24 This was the case in deliberations in Somerville and Boston (Barett 1981). The City of Boston was informed by legal experts that calling for a “moratorium” would be of “questionable legality” and leave the City open to court challenges that would run months or years, during which the ordinance could not take effect. “Report on Ordinance Regulating the Use of Recombinant DNA Technology. *Reports of Proceedings of the City Council of Boston* (20 May 1981), p. 282.

25 *Arthur D. Little, Inc. v. Com’r of Health,* 481 N.E.2d 441 (Mass. 1985).

26 The third major firm to anchor the early GBA biotechnology industry, Genzyme, was not part of these early discussions because its core products were based on enzyme purification, not rDNA (Yi 2009, pp. 79–80).

27 Sally Hughes interview with Thomas Kiley, Genentech Legal Counsel, conducted in 2000 and 2001, Regional Oral History Office, The Bancroft Library, University of California Berkeley, pp. 30–31. https://oac.cdlib.org/view?docId=kt9g5015sn&query=&brand=calisphere Accessed 21 February, 2024.

28 Introductory Comments by Professor Kenneth Murray (University of Edinburgh), Biogen Limited European Microbiology Conference, February 18-20, 1978. From papers provided by Philip Sharp. This structure was also reported in the business press (Powledge 1983, p. 400).

29 Minutes of Board of Supervisory Directors of Biogen NV, 14 January 1980, item #85. Cold Spring Harbor Laboratory Archives, Walter Gilbert Papers, Series 3, Box 5, Biogen 1980 (hereafter, Gilbert Papers).

30 A.J. Muller [director finance and admin, Biogen SA Geneva] “Biogen Inc. Research and Administration” memo to Cambridge Committees on Environmental Safety and Biohazards, 28 October 1980. Cambridge Public Health Department, rDNA Collection, Box 1, Biogen 1980-2002.

31 A.J. Muller (Biogen SA, Geneva) to R.E. Crawthorne and J.E. Davies, “US Laboratory Current Status,” 9 September 1980. Cold Spring Harbor Laboratory Archives, Charles Weissmann Papers Series 2, Box 19, “Biogen Board of Director Documents September 1980.”

32 Donald P. Dressler [Acting Commissioner CPHC] to James Sullivan [City Manager], 11 May 1979. Cambridge Public Health Department, Melvin Chalfen Papers, Cambridge Biohazard Comm.

33 Cambridge City Council Minutes, 20 October 1980 and 3 November 1980.

34 Comments of Biogen President, December 9, 1980 CERB Meeting with Biogen, Transcript. MIT Archives and Distinctive Collections, AC-502, Series 3, Box 4.

35 December 9, 1980, CERB Meeting with Biogen, Transcript. MIT Archives and Distinctive Collections, AC-502, Series 3, Box 4.

36 December 9, 1980, CERB Meeting with Biogen, transcript. MIT Archives and Distinctive Collections, AC-502, Series 3, Box 4.

37 Gabriel Schmergel Interview (Science History Institute Oral History #1035), p. 9. Hereafter, Schmergel Interview.

38 Ordinance for Regulating the Use of Recombinant DNA Technology (docket # 368) offered by Councilor Raymond Flynn. *Reports of Proceedings of the City Council of Boston*, 25 March 1981, pp. 91, 104.

39 “Report on Ordinance Regulating the Use of Recombinant DNA Technology.” *Reports of Proceedings of the City Council of Boston*, 20 May 1981, pp. 281–282.

40 Schmergel Interview, p. 29.

41 CCC 1976 Hearings Transcript, p. 57.

42 Prepared remarks of Frederick A. Eustis (Biogen Legal Counsel) for a Cambridge Biohazards Committee public hearing on Biogen Application, 13 May 1982. Cambridge Public Health Department, rDNA Collection, Box#1, Biogen 1980–2002.

43 Schmergel Interview, pp. 29–30.

44 Peter Feinstein Interview by author, 16 July 2019, Porter Square Books, Cambridge Mass.

45 “Memo: Massachusetts Section of IBA,” D. Glass [Biotechnica International] to R. Hardy, 21 November 1984; David Glass Personal Papers.

46 CERB Meeting Transcript, 6 January 1981, MIT Archives and Distinctive Collections AC-502, Series 3, Box 4.

47 Massachusetts Biotechnology Council “Biotechnology Regulatory Guide for Communities” (July 1995), p.5. David Glass Personal Papers

48 “Biotechnology Regulatory Guide for Communities,” p.13.

49 “Biotechnology Regulatory Guide for Communities,” p.19.

50 Comments of Biogen President, December 9, 1980, CERB Meeting with Biogen, Transcript. MIT Archives and Distinctive Collections, AC-502, Series 3, Box 4.

51 Don Megathin [assistant to Worchester Foundation President] testimony and prepared statement pp. 69-76, in Congressional Record 1994. See also Schmergel Interview, p. 30.

52 Minutes of the CBC [unapproved], 10 December 1985, Cambridge Public Health Department, Melvin Chalfen Papers [unprocessed], “Cambridge Biohazards Comm.”

53 From a 1998 10-K statement by Alexandria Real Estate, a firm that specializes in biotechnology space: “We and our tenants must comply with federal, state, and local laws and regulations that govern the use, manufacture, storage, handling and disposal of hazardous materials and waste products. Changes in these laws and regulations could adversely affect our business or our tenants' business and their ability to make rental payments to us.” https://are.gcs-web.com/static-files/71fe6b74-9656-49e7-99c9-35ec0b550fad

54 Interview with Peter Abair via Zoom by author, 5 March 2024. Also see Robert Coughlin [MBC President] to Brian Connors [Deputy Director Economic Development, Springfield, Mass.], 5 May 2009; available at https://www.springfield-ma.gov/planning/fileadmin/Planning_files/forms/BioReady_Ratings.pdf (Accessed June 10, 2024)

55 Revere City Council Meeting, 27 June 2022 (https://www.youtube.com/watch?v=bZrpurElVMU, accessed April 2, 2024).

56 Minutes of Coalition for Responsible Genetics Research, 7 May [1978-inferred from reference to other papers in folder] Tufts University Archives, Sheldon Krimsky Papers, Box 2, “Council for Responsible Genetics,” Folder 3/3. I read this with the critiques of how science studies scholars select the topics they study in relationship to hype and speculation by Lee Vinsel in mind, as cited above.
